# SARS-CoV-2 infection predisposes patients to coinfection with *Staphylococcus aureus*

**DOI:** 10.1128/mbio.01667-24

**Published:** 2024-07-22

**Authors:** Ashira Lubkin, Lucie Bernard-Raichon, Ashley L. DuMont, Ana Mayela Valero Jimenez, Gregory G. Putzel, Juan Gago, Erin E. Zwack, Olufolakemi Olusanya, Kristina M. Boguslawski, Simone Dallari, Sophie Dyzenhaus, Christin Herrmann, Juliana K. Ilmain, Georgia L. Isom, Miranda Pawline, Andrew I. Perault, Sofya Perelman, William E. Sause, Ifrah Shahi, Amelia St. John, Rebecca Tierce, Xuhui Zheng, Chunyi Zhou, Maria G. Noval, Anna O'Keeffe, Magda Podkowik, Sandra Gonzales, Kenneth Inglima, Ludovic Desvignes, Sarah E. Hochman, Kenneth A. Stapleford, Lorna E. Thorpe, Alejandro Pironti, Bo Shopsin, Ken Cadwell, Meike Dittmann, Victor J. Torres

**Affiliations:** 1 Department of Microbiology, New York University Grossman School of Medicine, New York, New York, USA; 2 Division of Gastroenterology and Hepatology, Department of Medicine, University of Pennsylvania Perelman School of Medicine, Philadelphia, Pennsylvania, USA; 3 Antimicrobial-Resistant Pathogens Program, Microbial Genomics Core Lab, New York University Langone Health, New York, New York, USA; 4 Department of Population Health, New York University Grossman School of Medicine, New York, New York, USA; 5 Department of Medicine, NYU Grossman School of Medicine, New York, New York, USA; 6 Division of Comparative Medicine, New York University Langone Health, New York, New York, USA; 7 High Containment Laboratories, Office of Science and Research, NYU Langone Health, New York, New York, USA; University of Illinois Chicago, Chicago, Illinois, USA

**Keywords:** COVID, MRSA, coinfection, *agr*, SARS-CoV-2

## Abstract

Severe COVID-19 has been associated with coinfections with bacterial and fungal pathogens. Notably, patients with COVID-19 who develop *Staphylococcus aureus* bacteremia exhibit higher rates of mortality than those infected with either pathogen alone. To understand this clinical scenario, we collected and examined *S. aureus* blood and respiratory isolates from a hospital in New York City during the early phase of the pandemic from both SARS-CoV-2+ and SARS-CoV-2− patients. Whole genome sequencing of these *S. aureus* isolates revealed broad phylogenetic diversity in both patient groups, suggesting that SARS-CoV-2 coinfection was not associated with a particular *S. aureus* lineage. Phenotypic characterization of the contemporary collection of *S. aureus* isolates from SARS-CoV-2+ and SARS-CoV-2− patients revealed no notable differences in several virulence traits examined. However, we noted a trend toward overrepresentation of *S. aureus* bloodstream strains with low cytotoxicity in the SARS-CoV-2+ group. We observed that patients coinfected with SARS-CoV-2 and *S. aureus* were more likely to die during the acute phase of infection when the coinfecting *S. aureus* strain exhibited high or low cytotoxicity. To further investigate the relationship between SARS-CoV-2 and *S. aureus* infections, we developed a murine coinfection model. These studies revealed that infection with SARS-CoV-2 renders mice susceptible to subsequent superinfection with low cytotoxicity *S. aureus*. Thus, SARS-CoV-2 infection sensitizes the host to coinfections, including *S. aureus* isolates with low intrinsic virulence.

**IMPORTANCE:**

The COVID-19 pandemic has had an enormous impact on healthcare across the globe. Patients who were severely infected with SARS-CoV-2, the virus causing COVID-19, sometimes became infected with other pathogens, which is termed coinfection. If the coinfecting pathogen is the bacterium *Staphylococcus aureus*, there is an increased risk of patient death. We collected *S. aureus* strains that coinfected patients with SARS-CoV-2 to study the disease outcome caused by the interaction of these two important pathogens. We found that both in patients and in mice, coinfection with an *S. aureus* strain lacking toxicity resulted in more severe disease during the early phase of infection, compared with infection with either pathogen alone. Thus, SARS-CoV-2 infection can directly increase the severity of *S. aureus* infection.

## INTRODUCTION

The coronavirus SARS-CoV-2, and the disease it causes, COVID-19, has resulted in an enormous amount of morbidity and mortality globally since late 2019. The clinical course of many viral infections can be complicated by subsequent bacterial or fungal infections, known as coinfections (or superinfections or secondary infections), which can lead to more severe disease (1, 2). This is also true for SARS-CoV-2 infection, in hospitalized patients. While the incidence of coinfections among critically ill COVID-19 patients is often reported to be around 15%, post-mortem studies suggest that the true incidence may be twice as high (3, 4). These discrepancies can be explained by limitations in the sensitivity of culture-based testing and the lack of adjunctive tests with high specificity, which hampers the ability to comprehensively track coinfections.

Nosocomial coinfections during COVID-19 can occur from many different pathogens. Bacterial pulmonary coinfections are mainly caused by Gram-negative bacteria and are associated with an extended duration of mechanical ventilation (5, 6). Pulmonary fungal coinfections have also been reported, particularly aspergillosis (7, 8). Bloodstream infections are reported for Gram-positive species, predominantly Staphylococcal and Enterococcal, as well as various Gram-negative species, albeit less frequently (9, 10). Bloodstream infections with fungal species have also been reported, notably from *Candida auris* and *Candida albicans* (11).

The threat of nosocomial *S. aureus* coinfection in COVID-19 patients has been recognized (12, 13). When compared with other pathogens, bloodstream coinfection with *S. aureus* has been shown to be associated with increased mortality (9, 14, 15), suggesting that SARS-CoV-2 may predispose patients to more severe *S. aureus* infection.

*S. aureus* pathogenesis is complex—the bacterium employs a multitude of virulence factors that can cripple the immune response and facilitate infection (16, 17). An important class of these virulence factors is the beta-barrel pore-forming cytotoxins, which include α-toxin and the bi-component leukocidins (18, 19). These toxins lyse phagocytes and many other cell types by forming pores in host cell membranes and, as such, are necessary for full virulence in several models of disease.

*S. aureus* is a notorious culprit of nosocomial infections, often with lethal consequences (20, 21). However, *S. aureus* isolates from nosocomial infections often exhibit low cytotoxic activity in tissue culture models using human neutrophils (22–24), implying lower production of cytotoxins. Inpatients are thought to be more susceptible to low-cytotoxicity strains because they often have medical devices in place and/or compromised immune systems. Underscoring this, isolates from the highly cytotoxic, community-associated lineage USA300, which have infiltrated hospitals (25–27), have been progressively losing their cytotoxic activity over a span of a few years (28).

To study *S. aureus* coinfections of SARS-CoV-2-infected patients, we collected *S. aureus* bloodstream and respiratory isolates from a hospital in New York City during the early phase of the COVID-19 pandemic. The *S. aureus* isolates were collected from both SARS-CoV-2+ and SARS-CoV-2- patients, and genotypic and phenotypic analyses were performed on all isolates. These studies revealed that clinical isolates from both SARS-CoV-2+ and SARS-CoV-2− patients exhibited broad phylogenetic and phenotypic diversity, with no significant phenotypic differences between the two patient groups. However, when focusing on bloodstream-infecting isolates, we found a trend toward lower cytotoxicity in the isolates recovered from SARS-CoV-2+ patients. To model the patient data and explore the SARS-CoV-2/*S. aureus* dynamic further, we developed a murine SARS-CoV-2/*S. aureus* coinfection model. Studies *in vivo* revealed that infection with SARS-CoV-2 worsens *S. aureus* disease severity and renders mice more susceptible to subsequent systemic infection by low-virulence *S. aureus*. Thus, SARS-CoV-2 infection leads to increased susceptibility to *S. aureus* coinfection, which helps shed light on the epidemiological connection between these two deadly pathogens.

## RESULTS

### Establishment of a biorepository of *S. aureus* during the early COVID-19 pandemic

**Fig 1:**
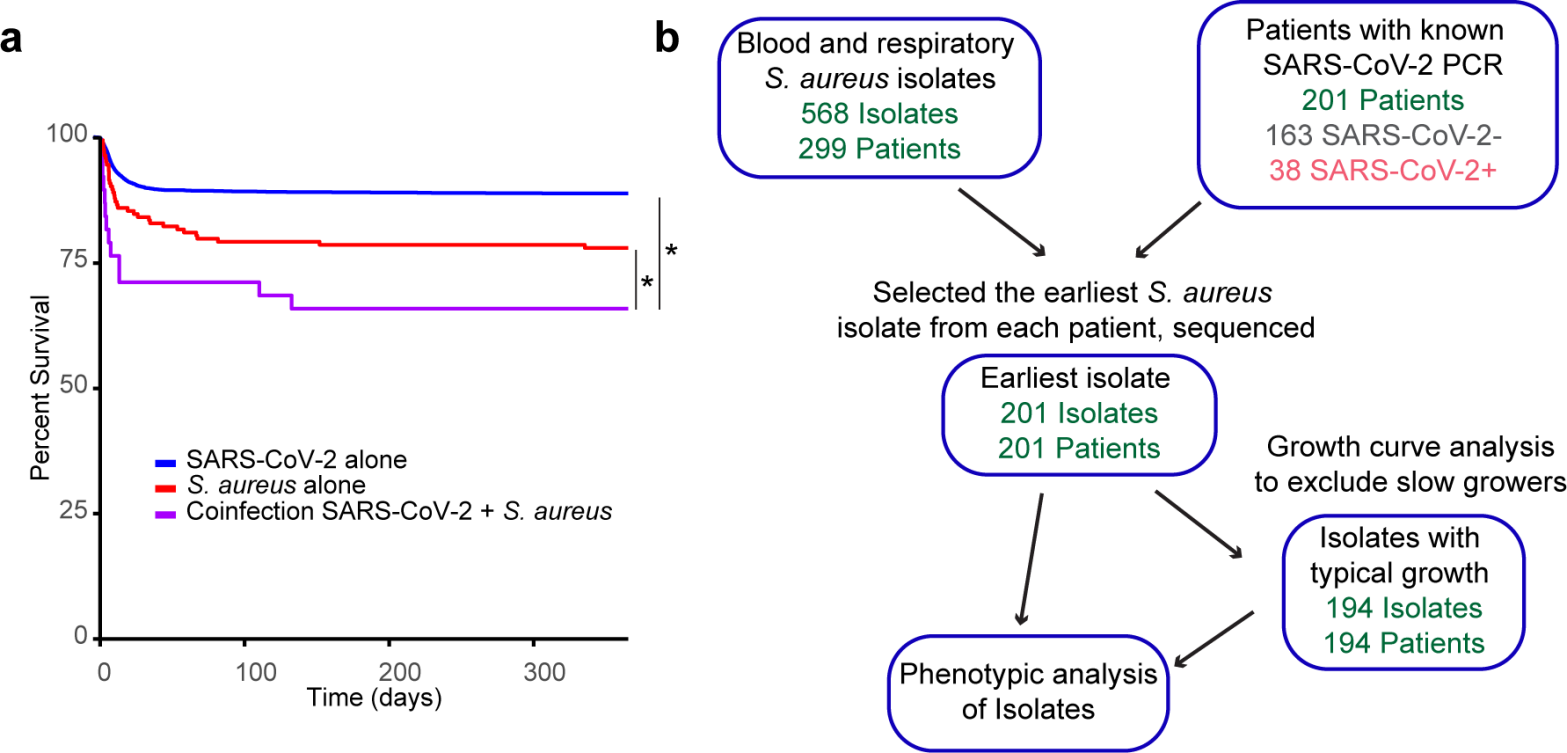
Establishment of a biorepository of *S. aureus* during the early COVID-19 pandemic (a) Survival curve of patients in our cohort with SARS-CoV-2 confirmed by PCR, with *S. aureus* blood or respiratory culture or with both infections. Time = 0 on the day of positive *S. aureus* culture or in the case of SARS-CoV-2 alone, on the day of SARS-CoV-2 PCR positivity. Data were analyzed by Wilcoxon-Breslow test. (b) Generation of the cohort of patients and isolates analyzed in this study. *P < 0.05

Coinfections can have an impact on patient outcomes in COVID-19, particularly those with *S. aureus*, which are associated with high mortality. This has been reported (9) and is observed within our cohort analyzed here (Fig. 1a). During the early phase of the COVID-19 pandemic, we began collecting coinfecting isolates from the NYU Langone Health Tisch Hospital clinical microbiology lab in late March 2020. We collected *S. aureus* clinical isolates from blood and respiratory cultures of both SARS-CoV-2 PCR+ and PCR− patients, which included isolates from bronchoalveolar lavage and expectorated sputum samples.

We focused on *S. aureus* isolates from the “first wave” of SARS-CoV-2 infection in New York City, which we defined as ending on August 31st, 2020, based on the subsiding incidence of SARS-CoV-2 in the NYU Langone Health system at that point in time. Bloodstream and respiratory isolates obtained from both sputum and bronchoscopy specimens were included in the analysis. As shown in Fig. 1b, this yielded 568 *S*. *aureus* isolates from 299 patients. We confined our *in vitro* phenotypic analysis to *S. aureus* isolates that we were able to link to patients with a recent SARS-CoV-2 PCR test and, if there were longitudinal samples, further restricted the analysis to the first *S. aureus* isolate recovered from each patient. This resulted in 163 patient-isolate pairs from SARS-CoV-2− patients and 38 patient-isolate pairs from SARS-CoV-2+ patients (Fig. 1b).

### Patients with SARS-CoV-2 became coinfected with diverse *S. aureus* strains

**Fig 2:**
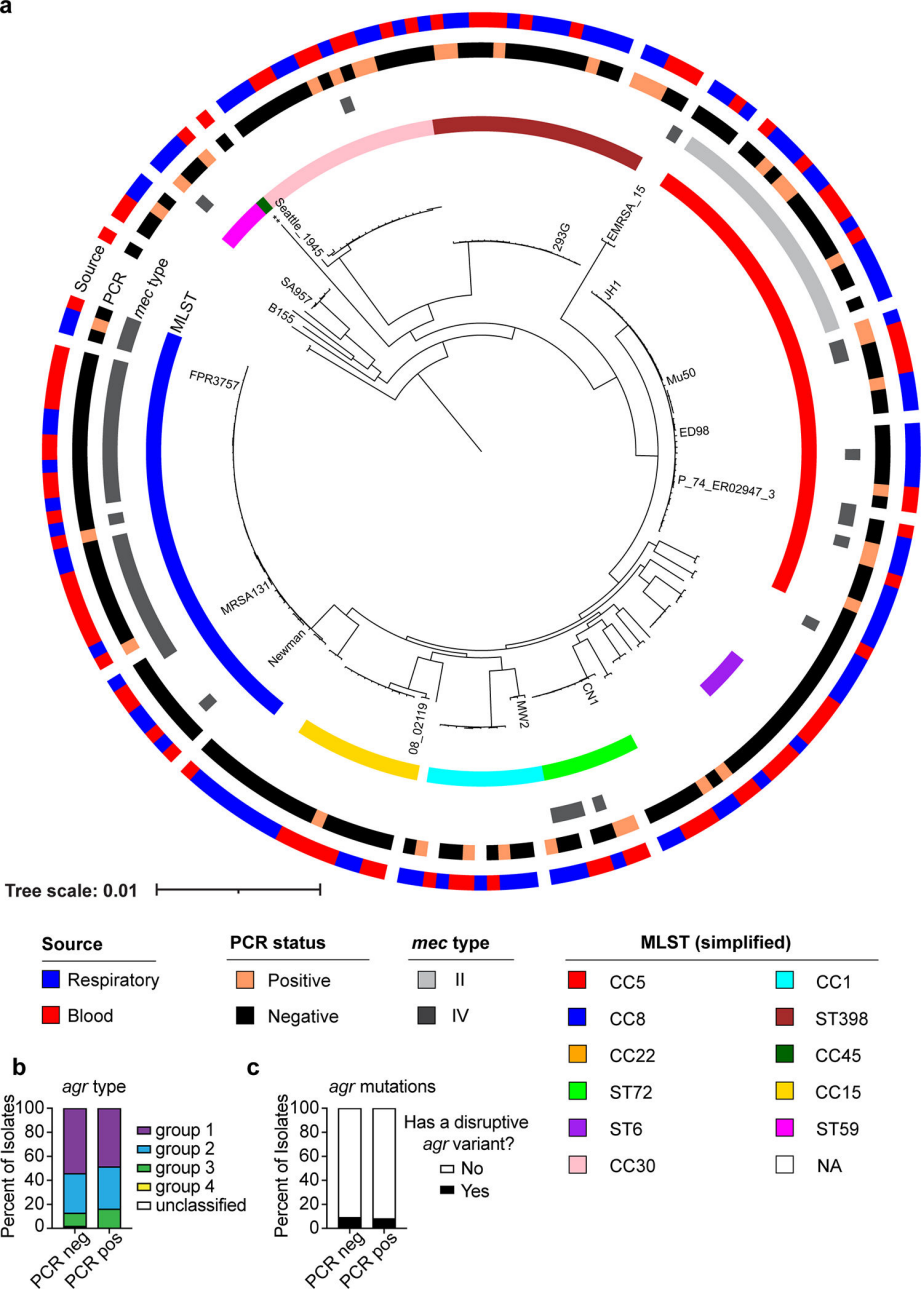
Phylogenetic analysis of *S. aureus* isolates. (a) The first isolate for each patient in the biorepository is included. Reference strains are labeled. Concentric circles represent CC/ST, *mec* type (only types II and IV were represented in this collection of isolates; if no *mec* type is indicated, the strain is *mec* negative), SARS-CoV-2 PCR result, and isolate source. (b) *agr* type and (c) *agr* mutations analyzed by SARS-CoV-2 PCR result. A disruptive *agr* variant was defined as a frameshift or stop gain. ** = reference strain CA-347. CC = clonal complex, ST = sequence type, “NA” = other or not categorized.

We performed whole-genome sequencing of all the *S. aureus* strains in our collection and found a wide range of phylogenetic diversity. The data for the constrained analysis on the first *S. aureus* isolate from each patient with a confirmed SARS-CoV-2 PCR test is depicted in Fig. 2, and the analysis for all the *S. aureus* isolates in the collection can be found in Fig. S1. The phylogenetic diversity is evident in the isolates from both SARS-CoV-2+ and SARS-CoV-2− patients. Indeed, there were no *S. aureus* clonal complexes or sequence types that were over-represented in the SARS-CoV-2+ isolate collection relative to the SARS-CoV-2− collection. Both collections had strong representation from the classically healthcare-associated clonal complexes CC5 and CC30, the classically community-associated (but increasingly healthcare-associated) clonal complex CC8, and the classically livestock-associated sequence type ST398 (Fig. 2a). In addition, both blood and respiratory isolates were well-represented throughout the phylogenetic tree for both the SARS-CoV-2+ and the SARS-CoV-2− isolate collections (Fig. 2a). The *mec* types that were represented for the methicillin-resistant *S. aureus* (MRSA) strains in this collection were types II and IV, although most isolates were methicillin-sensitive *S. aureus* (MSSA; *mec*-negative).

One of the major regulators of virulence in *S. aureus* is the accessory gene regulator, or Agr, a quorum-sensing system that controls the production of virulence factors, including toxins (29). One or more of the *agrBDCA* genes are frequently mutated in clinical isolates from the nosocomial setting (30), resulting in lower toxin production and, consequently, lower cytotoxicity to neutrophils and other immune cells (22). As shown in Fig. 2b, several *agr* types were present in our collection, and the distribution was similar between the SARS-CoV-2+ and SARS-CoV-2− patient isolates. To further analyze the incidence of *agr* mutations, we counted mutations resulting in frameshifts or premature stop codons as “disruptive” variants. We found that about 10% of the collection harbored disruptive variants in both groups (Fig. 2c). Thus, the overall genetic diversity of the *S. aureus* isolates is similar between the SARS-CoV-2+ and SARS-CoV-2− isolate collections.

### *S. aureus* strains have comparable growth, hemolysis, and cytotoxicity when isolated from patients with or without SARS-CoV-2 infection

Next, we performed phenotypic characterization of the isolates. We first analyzed their growth in rich medium (Tryptic Soy Broth; TSB) and found that while most of the strains grew at a similar rate to each other, about 3% were deficient in growth, never reaching an OD_600_ of 1 during overnight culture (Fig. S2a). These isolates were all from SARS-CoV-2− patients. We found that the isolates from patients with or without SARS-CoV-2 infection exhibited comparable maximum growth rate, lag time, and time to reach the stationary phase (Fig. S2b).

The ability to lyse red blood cells is an important virulence trait of *S. aureus*, since this is the most efficient way for the bacterium to acquire iron during infection (31). Thus, we tested the hemolytic activity of the isolates using blood agar plates. We found a range of alpha and beta hemolysis phenotypes in isolates from both SARS-CoV-2+ and SARS-CoV-2− patients (Fig. S2c). Overall, the hemolytic activity was similar between the two isolate collections, although the percentage of non-hemolytic isolates trended higher with SARS-CoV-2+ isolates compared with SARS-CoV-2− isolates (~35% vs ~20%; Fig. S2c).

*S. aureus* employs a prototypical yellow pigment to survive oxidative stress (32). Thus, we analyzed the color of the isolates when patched on TSA plates. We found a range of colors, with most isolates displaying a typical light-yellow pigment, whereas some were dark yellow, and some were white. However, there was no noticeable difference in pigment between isolates from SARS-CoV-2+ or SARS-CoV-2− patients (Fig. S2d).

**Fig 3:**
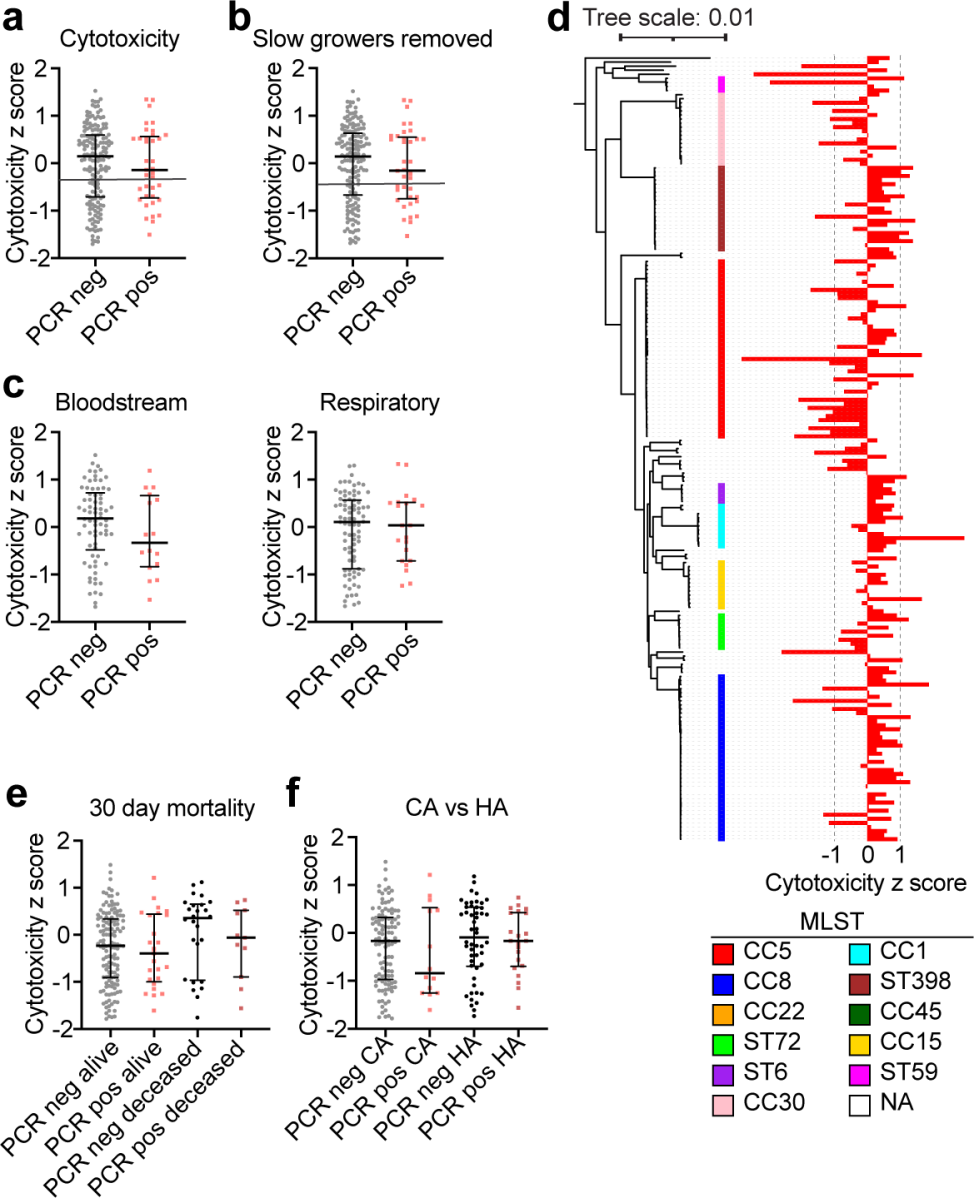
Analysis of cytotoxicity of *S. aureus* isolates *in vitro*. (a) Cytotoxicity z-score for each isolate. Cytotoxicity was analyzed by infecting PMNs from human donors and quantifying cell lysis via LDH release. PCR pos are isolates from SARS-CoV-2+ patients, and PCR− are isolates from SARS-CoV-2- patients. (b) As in panel a, but without the isolates that grew slowly and were not able to reach an OD_600_ of 1 by the end of the overnight growth. The line in a and b indicates the cutoff used to define high and low cytotoxicity strains. (c) Cytotoxicity z-sores separated by the site of isolation. (d) Cytotoxicity visualized across the phylogenetic tree. (e) Cytotoxicity of isolates analyzed by 30-day mortality of the patients. (f) Cytotoxicity of isolates analyzed by community (CA) vs. healthcare (HA) acquisition. Error bars represent median with interquartile range. *P* > 0.05 for all plots, Mann-Whitney test (a-c), Kurksal-Wallis test (e and f).

*S. aureus* is adept at evading the host immune response, and a major virulence strategy that it uses to kill neutrophils and other leukocytes is the secretion of pore-forming leukocidins (19, 33). To evaluate the cytotoxicity of the strains in our collection, we utilized a co-culture assay. In brief, *S. aureus* was grown overnight to the stationary phase and then used to infect primary human neutrophils for 2 h, after which time cell lysis was measured. Initially, we performed the analysis on all 201 *S*. *aureus* isolates in the collection and found that despite the isolates from SARS-CoV-2+ patients trending toward lower cytotoxicity, there was no statistically significant difference in cytotoxicity between isolates from SARS-CoV-2+ and SARS-CoV-2− patients (Fig. 3a). As noted above (Fig. S2a), about 3% of the strains did not reach an OD_600_ of 1 after overnight culture. Thus, we considered the possibility that these slow-growing strains would not be at a high enough density to produce leukocidins, as these toxins are produced upon quorum sensing (33). As a result, these strains would appear less cytotoxic simply because of their slow growth. To address this possibility, we reanalyzed the data with 194 isolates, having excluded strains that did not reach the cutoff of OD_600_ = 1 (Fig. 1b). We again found that there was no significant difference between the cytotoxicity of the isolates from SARS-CoV-2+ and SARS-CoV-2− patients (Fig. 3b). We used a Gaussian Mixture Model to define isolates as high or low cytotoxicity. The cutoff that was used based on this model is illustrated as a line in Fig. 3a and b.

To further explore the cytotoxicity data, we compared the isolates from blood cultures with those from respiratory samples. This analysis revealed that overall, both high- and low- cytotoxicity isolates were equally recovered from the blood and respiratory tract (Fig. 3c). We also plotted the cytotoxicity data in the context of phylogeny and found that the CC5 and CC30 strains displayed lower cytotoxicity, whereas the CC8, ST398, and CC1 strains displayed higher cytotoxicity (Fig. 3d), which is consistent with prior reports (23, 34). In addition, we compared the cytotoxicity of isolates from patients who survived at 30 days post-infection versus those who had died and found no significant differences in the cytotoxicity of isolates from these groups (Fig. 3e). Finally, we compared the cytotoxicity of strains acquired in the community with those that were healthcare-acquired (defined as infection after 48 h in the hospital) and found no significant differences in cytotoxicity (Fig. 3f). Overall, we did not find any statistically significant differences in the cytotoxicity of the isolates based on the SARS-CoV-2 status of the patient from whom they were isolated.

### Comparing the demographic and clinical characteristics of the patients

**Fig 4:**
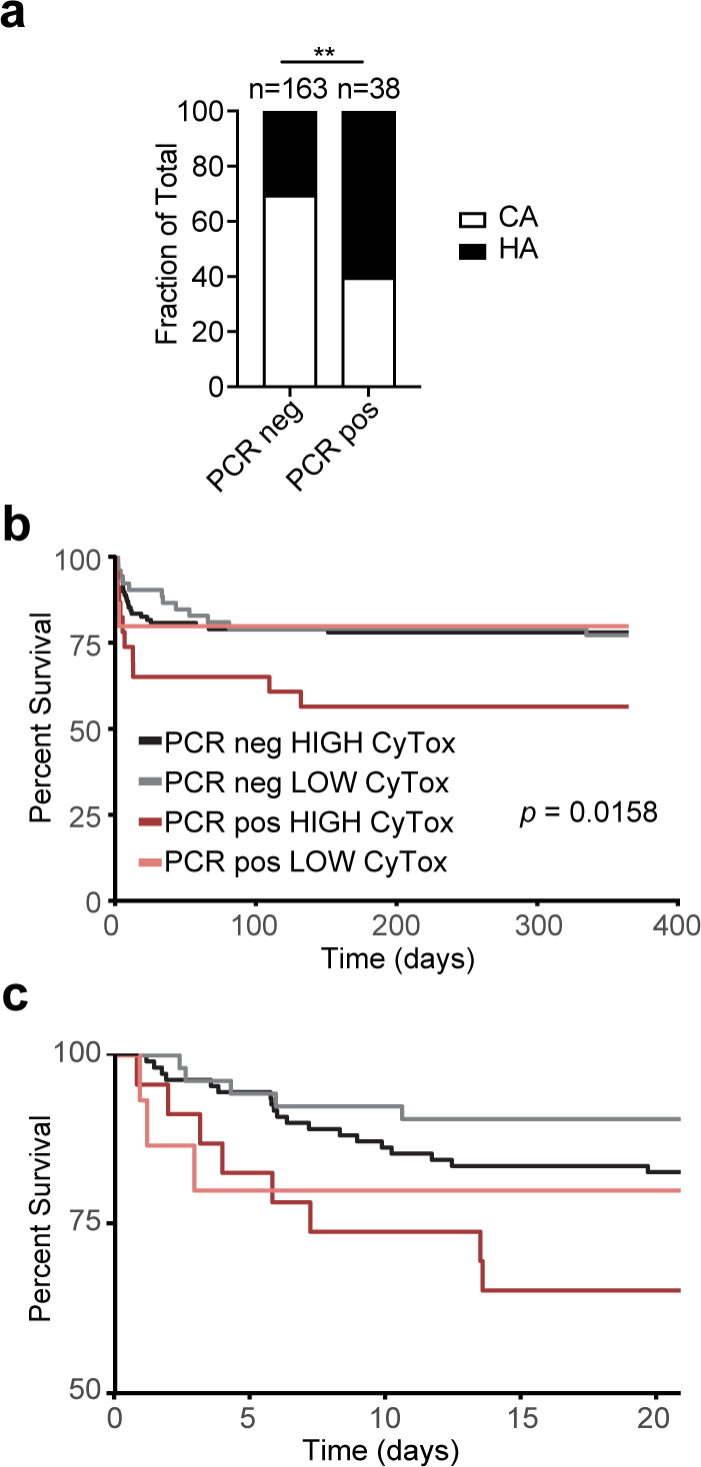
Patient data analysis. (a) Proportions of patients with community-acquired (CA) and healthcare-acquired (HA) *S. aureus* infection categorized by SARS-CoV-2 PCR result. (b) Survival curve of patients in the four groups described in Table 1. CyTox = cytotoxicity. (c) The same data as in b, focusing on the first 20 days post*-S. aureus* infection. **P < 0.01, c, Wilcoxon-Breslow test.

While we did not find a statistically significant difference in the cytotoxicity of the examined isolates, we nevertheless detected a trend where 50% of isolates from the blood cultures of SARS-CoV-2+ patients exhibited low cytotoxicity, compared with only 35% from SARS-CoV-2− patients (Fig. 3c). These data prompted us to further examine the cytotoxicity data in the context of patient demographics. The characteristics of the patient cohort whose isolates were analyzed here are summarized in Table 1. Since the cytotoxicity of isolates has been shown to correlate with patient outcomes (23), we broke down the cohort by cytotoxicity category—low or high, as well as by SARS-CoV-2 PCR test result. All four patient groups in Table 1 were majority male, consistent with many reports that severe COVID-19 is more common in males (35), but the racial/ethnic spread was similar between groups. For the SARS-CoV-2- patients (PCR neg), about two-thirds of the isolates were community-acquired and one-third were hospital-acquired (Table 1). This was different in the SARS-CoV-2+ group (PCR pos), where the low-cytotoxicity group originated equally from the community and the hospital and the high-cytotoxicity group originated mostly from the hospital (Table 1). Indeed, analyzing the infection origin by SARS-CoV-2 status alone, we observed that the SARS-CoV-2+ patients had more hospital-acquired *S. aureus* infections than the SARS-CoV-2− patients (Fig. 4a).

**TABLE 1:** Patient demographics and clinical characteristics^*a*^

	PCR neg high	PCR neg low	PCR pos high	PCR pos low	
	CyTox (*N* = 110)	CyTox (*N* = 53)	CyTox (*N* = 23)	CyTox (*N* = 15)	*P* value
Sex					0.986
Female	36 (32.7%)	16 (30.2%)	7 (30.4%)	5 (33.3%)	
Male	74 (67.3%)	37 (69.8%)	16 (69.6%)	10 (66.7%)	
Race/ethnicity					0.567
Asian	7 (6.4%)	3 (5.7%)	4 (17.4%)	1 (6.7%)	
Black	14 (12.7%)	5 (9.4%)	0 (0.0%)	2 (13.3%)	
Other/multiracial	36 (32.7%)	16 (30.2%)	9 (39.1%)	4 (26.7%)	
White	53 (48.2%)	29 (54.7%)	10 (43.5%)	8 (53.3%)	
Nosocomial, >48 h					0.002
Community	74 (67.3%)	39 (73.6%)	7 (30.4%)	8 (53.3%)	
Nosocomial	36 (32.7%)	14 (26.4%)	16 (69.6%)	7 (46.7%)	
Length of stay (days)					0.122
Mean (SD)	20.56 (47.85)	13.21 (13.95)	36.43 (35.79)	18.60 (16.85)	
Median (Q1, Q3)	10.00 (2.00, 22.00)	10.00 (2.00, 15.00)	19.00 (10.00, 57.50)	11.00 (7.50, 29.00)	
Mortality at 7 days					0.128
Deceased	13 (11.8%)	4 (7.5%)	6 (26.1%)	3 (20.2%)	
Mortality at 30 days					0.072
Deceased	21 (19.1%)	5 (9.4%)	8 (34.8%)	3 (20.0%)	
Mortality 1 year					0.159
Deceased	24 (21.8%)	12 (22.6%)	10 (43.5%)	3 (20.0%)	
Source					0.456
Blood	54 (49.1%)	20 (37.7%)	9 (39.1%)	8 (53.3%)	
Respiratory	56 (50.9%)	33 (62.3%)	14 (60.9%)	7 (46.7%)	
Age, years					0.319
Mean (SD)	53.51 (25.11)	57.28 (23.99)	55.70 (22.84)	65.33 (17.14)	
Median (Q1, Q3)	60.00 (39.00, 73.00)	62.00 (46.00, 75.00)	58.00 (36.50, 72.00)	70.00 (57.50, 73.00)	
Charlson comorbidity index					0.325
Mean (SD)	3.45 (3.34)	3.72 (3.40)	2.39 (2.74)	2.60 (3.33)	
Median (Q1, Q3)	2.50 (1.00, 5.00)	3.00 (1.00, 6.00)	2.00 (0.00, 3.50)	2.00 (0.00, 3.50)	
Comorbidities					
Cancer	27 (24.5%)	17 (32.1%)	4 (17.4%)	2 (13.3%)	0.357
Cerebrovascular disease	34 (30.9%)	17 (32.1%)	6 (26.1%)	2 (13.3%)	0.514
Congestive heart failure	23 (20.9%)	15 (28.3%)	1 (4.3%)	2 (13.3%)	0.104
Chronic pulmonary disease	41 (37.3%)	31 (58.5%)	3 (13.0%)	4 (26.7%)	0.001
Diabetes no complications	37 (33.6%)	21 (39.6%)	7 (30.4%)	7 (46.7%)	0.655
Diabetes with complications	18 (16.4%)	12 (22.6%)	2 (8.7%)	3 (20.0%)	0.498
Myocardial infarction	25 (22.7%)	13 (24.5%)	4 (17.4%)	4 (26.7%)	0.897
Mild liver disease	12 (10.9%)	3 (5.7%)	5 (21.7%)	2 (13.3%)	0.226
Mod/ severe liver disease	4 (3.6%)	2 (3.8%)	3 (13.0%)	0 (0.0%)	0.180
Peripheral vascular disease	24 (21.8%)	13 (24.5%)	1 (4.3%)	5 (33.3%)	0.135
Renal disease	40 (36.4%)	15 (28.3%)	3 (13.0%)	3 (20.0%)	0.111

^*a*^ Patients were divided into four groups: (i) patients with a negative SARS-CoV-2 PCR, with *S. aureus* isolates that were high cytotoxicity, (ii) patients with a negative SARS-CoV-2 PCR, with *S. aureus* isolates that were low cytotoxicity, (iii) patients with a positive SARS-CoV-2 PCR, with *S. aureus* isolates that were high cytotoxicity, and (iv) patients with a positive SARS-CoV-2 PCR, with *S. aureus* isolates that were low cytotoxicity. CyTox = cytotoxicity. Demographics and clinical characteristics of each group are shown.

The length of hospital stay also trended longer in the SARS-CoV-2+ group (Table 1), consistent with published findings (9), though not statistically significant. Mortality at 30 days and 1 year trended higher in the SARS-CoV-2+ patients coinfected with high cytotoxic strains. Age was similar across the groups, as was the incidence of comorbidities, making these factors unlikely contributors to the observed trend in mortality (Table 1).

We took a deeper look at the mortality in our cohort, which includes both patients with bloodstream and respiratory isolates. We found that patients coinfected by SARS-CoV-2 and highly cytotoxic strains of *S. aureus* were more likely to die than those in the other three groups (SARS-CoV-2+ with low-cytotoxicity *S. aureus*, and SARS-CoV-2- with either high- or low-cytotoxicity *S. aureus*) (Fig. 4b). Interestingly, this cytotoxicity-dependent mortality among coinfected patients reveals itself only after the first week post-*S. aureus* infection – in the first week, patients coinfected with a low cytotoxicity isolate did not die less than those coinfected with a high cytotoxicity isolate (Fig. 4c; Table 1). One way to interpret these findings is that patient mortality in the first week of SARS-CoV-2 infection was likely driven by the virus and the fact of coinfection, more than by the virulence attributes of the coinfecting *S. aureus* strain.

### SARS-CoV-2 infection sensitizes mice to *S. aureus* coinfection

**Fig 5:**
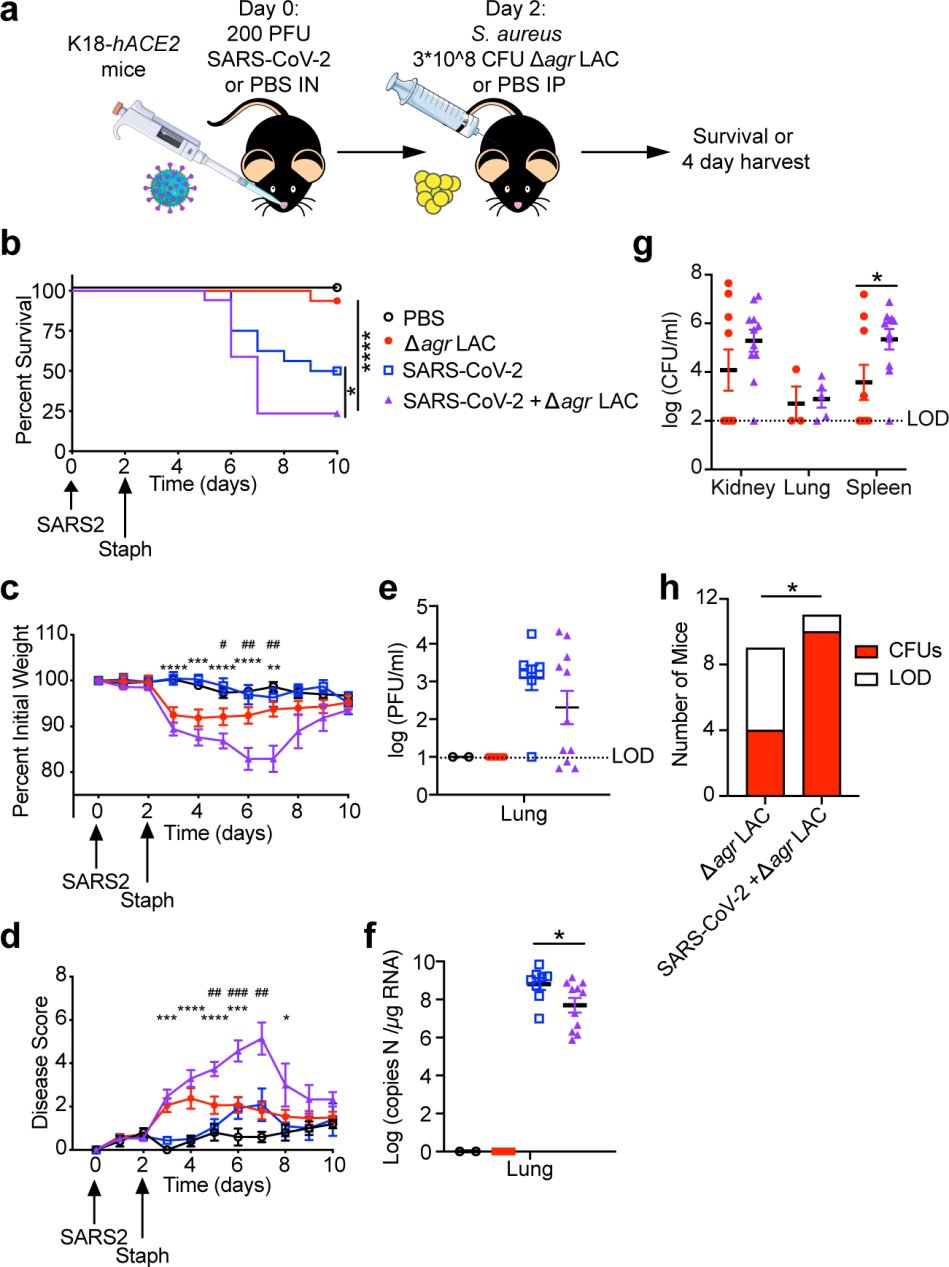
SARS-CoV-2 increases susceptibility to *S. aureus* in mice. (a) Schematic of coinfection experimental design. Survival (b), weight loss (c), and disease score (d) of mice infected with the indicated pathogens (15–17 mice per group, 5 mice in the PBS group). Viral burdens in lungs as determined by plaque assay (e), and qPCR (f) at 4 days post-infection. (g) Bacterial burdens of *S. aureus* in the indicated organs by CFU at 4 days post-infection. (h) Number of mice that were colonized with *S. aureus* by 4 days post-infection in any organ (CFUs) or not (LOD) (h). Error bars represent mean +/- SEM. *P < 0.05, **P < 0.01, ***P < 0.001, ****P < 0.0001. b, Mantel-cox test, c, d, Mann-Whitney test, g, Student’s *t*-test, h, Fisher’s exact test. In panels c and d, * indicates a difference between the SARS-CoV-2 infected group and the coinfected group,and ^#^ indicates a difference between the *S. aureus* infected group and the coinfected group.

Based on the finding that patients with SARS-CoV-2 and *S. aureus* coinfections have increased mortality compared to patients with SARS-CoV-2 or *S. aureus* infection alone (Fig. 1a), we developed a coinfection model in mice to study the dynamics of SARS-CoV-2 and *S. aureus in vivo* (Fig. 5a). We hypothesized that SARS-CoV-2 infection sensitizes the host to *S. aureus* and causes what would otherwise be a sublethal infection to become lethal. To test this, we used a Δ*agr* LAC, a USA300 strain of *S. aureus* (CC8), as *agr* mutations were observed in our contemporary collection of clinical isolates and *agr* mutants are associated with nosocomial infections. Moreover, using the Δ*agr* strain facilitated a sublethal infection as Δ*agr* strains produce fewer toxins than WT USA300 and are highly attenuated in mouse models (36). This was necessary to study the impact of SARS-CoV-2/*S. aureus* coinfection in mice, because it allowed us to develop a model whereby *S. aureus* on its own was not lethal. We used K18-*hACE2* mice as a model, in which the human *ACE2* gene is driven by the K18 promoter, rendering the mice susceptible to SARS-CoV-2, including to the ancestral SARS-CoV-2 isolate WA-1 (37), which was the circulating lineage during the time we collected *S. aureus* isolates.

The K18-*hACE2* mice were infected with a sub-lethal dose of SARS-CoV-2 WA-1 intranasally, and 2 days later, the mice were infected with *S. aureus* intraperitoneally to model systemic infection (Fig. 5a). In line with our patient data, we found that mice coinfected with *S. aureus* after SARS-CoV-2 infection exhibited greater morbidity and mortality than mice infected with either SARS-CoV-2 or *S. aureus* alone (Fig. 5b through d).

We next sought to determine the mechanism for this increased morbidity and mortality. We tested whether coinfection with *S. aureus* increased viral burden in the lungs but found no increase in either infectious viral particles (measured by plaque-forming units, PFU) or total viral particles (measured by copies of viral RNA) in the coinfected mice. In fact, the opposite trend was observed with less PFU and viral RNA in the lungs of SARS-CoV-2/*S. aureus* coinfected mice compared with SARS-CoV-2 infection alone (Fig. 5e and f). In contrast, the bacterial burden measured by colony-forming units (CFUs) in coinfected mice was significantly increased in the spleen compared with mice infected with *S. aureus* alone, and this trend was also observed in the kidneys and lungs of coinfected mice (Fig. 5g). More strikingly, we found that less than half of the mice singly infected with *S. aureus* had detectable bacterial burden in any of the organs examined 2 days post-*S. aureus* infection, whereas 90% of the coinfected mice had detectable *S. aureus* burden at this time point (Fig. 5h). We analyzed the extent of pathology of the lungs by histology and did not find any notable pathology in the sections of lung examined (Fig. S3). We also compared the serum levels of soluble mediators between the groups (Fig. S4). We found that serum cytokine levels were mostly driven by *S. aureus* infection, without significant differences between the *S. aureus*-infected and coinfected groups that could explain the survival difference that we observed. In sum, our data established that prior infection with SARS-CoV-2 predisposed mice to infection with low virulence *S. aureus*, resulting in a more severe bacterial infection and dissemination.

## DISCUSSION

In this work, we collected and analyzed *S. aureus* isolates from SARS-CoV-2+ patients during the first wave of the COVID-19 pandemic in a large metropolitan hospital in New York City. We found broad diversity in the collected isolates, both genetically and phenotypically, which is representative of the molecular epidemiology of *S. aureus* in the USA. Thus, the acquisition of *S. aureus* strains among the patients in this cohort did not represent a nosocomial outbreak of a single clone but rather the typical acquisition of *S. aureus* infections, both in the community and in the hospital.

Our phenotypic analyses did not reveal statistically significant differences between the isolates from SARS-CoV-2+ and SARS-CoV-2− patients. The majority of the isolates displayed high cytotoxicity, with a substantial minority showing low cytotoxicity in our *in vitro* co-culture system. It should be noted that a major limitation of this study is the imbalance in the number of isolates between the SARS-CoV-2+ and SARS-CoV-2− patient groups—the SARS-CoV-2− isolate collection had about four times the number of isolates of the SARS-CoV-2+ isolate collection. It is possible that a study with an increased number of clinical isolates would have sufficient statistical power to reveal differences between these two groups of isolates. Nevertheless, we observed that more of the bloodstream isolates from SARS-CoV-2+ patients had low cytotoxic and low hemolytic potential *in vitro*, compared with bloodstream isolates from SARS-CoV-2 negative patients.

We found that patients with SARS-CoV-2 and superinfecting bloodstream or respiratory *S. aureus* were more likely to die from infection. In the first few days post-infection, patient mortality did not correlate with the cytotoxicity of the infecting *S. aureus*, but after about a week, the coinfection with high-cytotoxicity *S. aureus* correlated with a higher risk of mortality. Our murine experiments, which were short-term and conducted with a low-cytotoxicity strain, modeled this initial acute phase of infection, where *S. aureus* increases patient mortality in a manner that seems to be independent of cytotoxic activity. An interesting direction for future work could be to model longer-term infections with cytotoxic strains. In practice, this would be difficult though, since mice are highly susceptible to cytotoxic strains of *S. aureus* and typically succumb to infection within the first week. Another important direction for future work is to study coinfection using clinical *S. aureus* isolates, especially those from SARS-CoV-2+ patients. It would be illuminating to compare naturally occurring low-cytotoxicity isolates with the *agr* mutant in this experimental system.

There are several possible explanations as to why SARS-CoV-2 predisposes patients to coinfections in general and in particular to severe infections with *S. aureus*. One possibility is that there is a direct interaction between the virus (and/or the immune response or microbiome disruption caused by the virus) and the coinfecting microbe. There are many potential mechanisms for this. For example, an *S. aureus* iron-binding protein has been shown to increase SARS-CoV-2 replication by modulating host transcription (38). SARS-CoV-2 infection also can impact the microbiome, promoting gut barrier disruption and translocation of microbes from the gut to the bloodstream (39). Thus, prior SARS-CoV-2 infection could overwhelm the immune system and render the host susceptible to superinfections.

A second possibility is that the treatments given to patients promote coinfection. Indeed, COVID-19 patients are often given corticosteroids, which dampen the immune system, and antibiotics, which promote the growth of fungi and resistant bacteria at mucosal sites (40, 41). Furthermore, early on during the COVID-19 pandemic, IL-6 inhibition was used as an investigational treatment (42), which disrupts the intestinal barrier (43), and has been associated with a higher incidence of coinfections in COVID-19 patients (44). This cocktail of treatments is the perfect recipe to promote bloodstream coinfections.

A third possibility is that the hospital environment during the pandemic promoted coinfections. Patients in hospitals are more frequently malnourished and dehydrated (45, 46), which impacts the immune response (47). It is possible that patients in respiratory isolation may be even more impacted by these factors.

Our *in vivo* mouse data support, at least partially, the first possibility—that the virus itself, or the host response to it, can directly increase COVID-19 patients’ susceptibility to systemic *S. aureus* infection. Whether the effect is caused directly by the virus or by the host immune system or microbiome is an important question for future studies. A recent study of pulmonary co-infection of SARS-CoV-2 and *Streptococcus pneumoniae* revealed not only higher bacterial and viral burdens in the coinfected group but also higher inflammatory mediators (48). In our model, we did not measure notable changes in the serum cytokine/chemokine levels (Fig. S4). Important goals for future studies of SARS-CoV-2/*S. aureus* coinfection should include an in-depth tissue-specific analysis of the inflammatory state during coinfection, including transcriptional analysis and cytokine levels in the bronchoalveolar lavage fluid. Of course, it is likely that a combination of factors promotes *S. aureus* infection in SARS-CoV-2+ patients. Treatments that COVID-19 patients received, and the hospital environment, could have additive effects upon those driven by the viral infection. Future studies are needed to investigate the combined effects of SARS-CoV-2 infection and antibiotic or steroid treatment or models of poor nutrition and hydration on coinfection with *S. aureus*.

Virus-*S. aureus* interactions are much older than the COVID-19 pandemic. Multiple respiratory viruses, including influenza and RSV, predispose patients to *S. aureus* and other bacterial co-infections (49–51). Several mechanisms have been shown to play roles in this process, including direct interactions between the virus and the bacteria, increased nutrient availability, alteration of immune cell functions and cytokine milieu, and microbiome derangements (2). How many of these mechanisms are at play during coinfection with SARS-CoV-2 and *S. aureus* is a fascinating topic for future research.

It should be stated here that this study does not argue for broadly applied antibiotic treatment for patients with SARS-CoV-2 without indication. Antibiotics do not improve outcomes in COVID-19 patients without evidence of coinfection (52). Increasing antibiotic resistance is a major healthcare threat, and the COVID-19 pandemic has expanded this problem (53–55). Furthermore, COVID-19 patients treated with broad-spectrum antibiotics upon admission actually had higher rates of coinfections (56). As always, responsible antibiotic stewardship is of paramount importance.

In sum, we have described a cohort of *S. aureus* isolates recovered from both SARS-CoV-2+ and SARS-CoV-2− patients during the early phase of the COVID-19 pandemic in New York City and uncovered a link between SARS-CoV-2 infection and increased *S. aureus* pathogenesis when SARS-CoV-2/*S. aureus* coinfection is modeled in mice. Furthermore, the development of this murine SARS-CoV-2/*S. aureus* coinfection model should facilitate future studies exploring the mechanisms driving the lethal impact of SARS-CoV-2/*S. aureus* coinfections.

## MATERIALS AND METHODS

### Biorepository banking and bacterial strains

*S. aureus* clinical isolates were acquired from the NYU clinical microbiology lab. All *S. aureus* isolated from blood and respiratory samples by the clinical lab between March 29, 2020, and August 31, 2020 were collected. They were streaked on MSA plates, cultured overnight (ON) at 37°C, and a single colony was patched on trypticase soy agar (TSA) plates, grown ON at 37°C, and then frozen at −80°C. Isolates were then streaked again on MSA or TSA with 5% sheep blood (Henry Schein), and three pooled colonies for each isolate were used to seed 96 well plates with TSB for ON growth and freezing. Frozen 96 well plates were stamped on TSA agar for high-throughput assays.

The USA300 AH1263 (WT AH-LAC; VJT # 15.77) (57) and Δ*agr* AH-LAC (VJT # 36.34) (58), and Δ*luk*Δ*hla* AH-LAC (VJT # 58.79) (59) strains were used as controls for all *in vitro* assays, and Δ*agr* AH-LAC was used for the coinfection of mice. All experiments involving recombinant *S. aureus* were performed according to protocols approved by the NYU Grossman School of Medicine Institutional Biosafety Committee (IBC21-000096-01, Torres).

### Genome sequencing, assembly, and annotation

The isolates were further purified from the frozen stocks by streaking on TSA with 5% sheep blood. Genomic DNA was extracted using the KingFisher Flex automated extraction instrument (Thermo Fisher) and the MagMAX DNA Multi-Sample Ultra 2.0 kit reagents (Applied Biosystems). Genome sequencing was performed on an Illumina NovaSeq 6000 device at the NYU Genome Technology Core, yielding 150 bp paired-end reads. This yielded a mean of 3.5 million read pairs per isolate (standard deviation: 1.1 million read pairs), corresponding to 1.05 Gbp (standard deviation: 0.37 Gbp) per isolate.

Raw reads were quality-filtered, trimmed, and stripped of adapters using fastp version 0.20.1 (60). The resulting reads were assembled into contigs using Unicycler version 0.4.8 (61) in conservative mode. Filtered, trimmed reads were then mapped to the assemblies using BWA 0.7.17 (62); isolates with mean depth below 100 were excluded from further analysis. Taxonomic classification of assembled genomes was performed using GTDBTK version 1.5.1 (63) using release 202 of the GTDB database (64). Only isolates whose assemblies were classified as *S. aureus* were further analyzed. Within-species (cross-strain) contamination was assessed using ConFindr version 0.7.4 (65), with isolates having estimated contamination greater than 10% excluded from further analysis. The *mecA* gene was detected and SCCmec types were determined using staphopia-sccmec (https://github.com/staphopia/staphopia-sccmec), part of the Staphopia pipeline (66).

Filtered and trimmed reads were mapped to a reference genome assembly of *S. aureus* strain FPR3757 (RefSeq accession number GCF_000013465.1) using Snippy version 4.6.0 (67); a core genome alignment was then generated using the Snippy command snippy-core. A phylogenetic tree was inferred from the resulting alignment using version 8.2.12 of RAxML (68) using the GTRGAMMA of nucleotide substitutions. Phylogenetic trees were visualized using the Interactive Tree of Life (iTOL (69)). The software AgrVATE v.1.0.2 (70) was used to detect variants in the *agr* gene and predict their effects.

### Growth curve, hemolysis, and color analysis of *S. aureus* isolates

Growth curve analysis was performed in TSB media using an Agilent BioTek LogPhase 600 Microbiology Reader. Isolates were each assayed three times and were designated as slow growers if the majority of their runs failed to reach an OD_600_ of 1.0. The curves of all three growth curves for each isolate are shown in Fig. S2a.

The color of the isolates was analyzed by taking pictures of the isolates stamped on TSA plates. The images were analyzed for % yellow using Photoshop in CMYK color mode, in order to quantify how yellow each isolate was. This was done three times for each isolate, and the % yellow was averaged.

The hemolytic capacity of the isolates was analyzed by patching them onto TSA plates with 5% sheep blood and growing overnight at 37°C. The plates were photographed and then placed a 4°C ON, and photographed again. Alpha-hemolysis was scored based on the original images before cold shock, in comparison to control lab strains AH-LAC, Δ*agr* AH-LAC, and Δ*luk*Δ*hla* AH-LAC. Beta-hemolysis was scored by comparing the images after cold shock to those before cold shock.

### Cytotoxicity analysis of *S. aureus* isolates

Primary human polymorphonuclear leukocytes (hPMNs) were isolated from LeukoPaks of human blood samples as previously described (71). In order to facilitate most of the isolates reaching a similar OD, hPMNs were infected with *S. aureus* isolates after overnight culture. Overnight culture was performed in 125 µL TSB in a 96-well round bottom plate, at 37°C in a shaker at 180 rpm. Overnight cultures were washed in PBS, resuspended in 200 µL PBS, and 20 µL was used to infect 200,000 hPMNs in 80 µL RPMI 1640 with 0.1% Human Serum Albumin (HSA) and 0.01M HEPES. This resulted in an MOI of approximately 200 for those strains that were able to reach OD = 1 during overnight culture (Fig. S2a). hPMNs and *S. aureus* were then synchronized by centrifuging 1,500 rpm for 7 min at RT to bring them into close contact. After a 2 h infection at 37°C and 5% CO_2_, hPMN viability was determined by LDH release (CytoTox-ONE Homogeneous Membrane Integrity Assay, Promega), measured using the PerkinElmer Envision plate reader.

The hPMN viability was determined for eight blood donors for each *S. aureus* isolate. Within each donor’s data, the raw fluorescence signals across all isolates were normalized by z scoring. Thus, each assayed isolate received a z score for each of the eight donors; the median of these is referred to as the cytotoxicity z score in the text.

The isolates were grouped into high- and low-cytotoxicity categories. The mixtools R package version 1.2.0 (72) was used to infer a Gaussian mixture model (GMM) with k = 2 components corresponding to high and low cytotoxicity. Specifically, the normalmixEM function was applied to the cytotoxicity z scores, fitting a GMM model and assigning to each isolate a posterior probability of belonging to the high-cytotoxicity group. Isolates were labeled "high cytotoxicity" if this posterior probability was greater than 0.5, and "low cytotoxicity" otherwise.

### Patient data analysis

Patients’ demographic and clinical data were extracted from Electronic Health Records (EHR) using structured SQL queries. Continuous variables were expressed as mean ± standard deviation (SD) or median with interquartile range (IQR). Discrete variables were presented as frequencies and percentages. Infections were classified into nosocomial and community-acquired. Nosocomial infections were those acquired after 48 h of admission, community-acquired infections were those identified within the first 48 h since admission. The study encompassed cases recorded from March 24, 2020, to August 31, 2020. For a comprehensive mortality comparison with all SARS-CoV-2 cases, all patients who tested PCR-positive during the same period were included in the study. Co-infections were identified through a combination of positive PCR tests for SARS-CoV-2 and confirmed cultures for *S. aureus*. A case was considered a coinfection if the SARS-CoV-2 test was performed 15 days before the positive *S. aureus* culture or if the virus was detected up to 11 days after the culture. We included patients where SARS-CoV-2 was detected after the *S. aureus* infection because in the early days of the COVID-19 pandemic, SARS-CoV-2 tests were often delayed.

### Viral culture and cell culture

Vero E6 cells (CRL-1586; American Type Culture Collection) were cultured in Dulbecco’s Modified Eagle’s Medium (DMEM, Corning) supplemented with 10% fetal bovine serum (FBS, Atlanta Biologics) and Penicillin/Streptomycin (Corning). SARS-CoV-2 isolate USA-WA1/2020, deposited by the Centers for Disease Control and Prevention, was obtained through BEI Resources, NIAID, NIH (cat. no. NR-52281; GenBank accession number MT233526). The stock was passaged in Vero E6 cells, and pooled medium was used to plaque purify a single virus clone on Vero E6 cells in the presence of 1  µg/mL l-1-tosylamido-2-phenylethyl chloromethyl ketone (TPCK)-trypsin to avoid virus adaptation to Vero E6 cells due to the lack of TMPRSS2 expression (65). Purified plaques were whole-genome sequenced to verify the presence of signature clade B amino acid changes, S D614G and NSP12 P323L, and the absence of furin cleavage site mutations, before expanding in the presence of TPCK-trypsin to generate a passage six working stock (5*10^5 PFU/mL) as described here (73). All SARS-CoV-2 stock preparations and subsequent infection assays were performed in

animal biosafety level 3 facility (ABSL3) of NYU Grossman School of Medicine (New York, NY), in accordance with its Biosafety Manual and Standard Operating Procedures. All experiments involving SARS-CoV-2 were performed according to protocols approved by the NYU Grossman School of Medicine Institutional Biosafety Committee (IBC IBC22-000088, Dittmann).

### Coinfection mouse model

Heterozygous K18-hACE2 C57BL/6 J mice (strain: 2B6.Cg-Tg(K18-ACE2)2Prlmn/J) were obtained from The Jackson Laboratory or bred in-house. Animals were housed in groups and fed standard chow diets. All animal studies were performed according to protocols approved by the NYU Grossman School of Medicine Institutional Animal Care and Use Committee (IACUC IA10-00071, Dittmann). Seventeen-week-old K18-hACE2 females were administered either 200 PFU SARS-CoV-2 diluted in 25 µL PBS (Corning) or mock-infected with 25 µL PBS via intranasal administration under xylazine-ketamine anesthesia (AnaSed AKORN Animal Health, Ketathesia Henry Schein Inc). Viral stocks were thawed, diluted to the working inoculum, and then stored at 4°C the day prior to infection. Viral titer in the inoculum was verified by plaque assay in Vero E6 cells. Two days post-SARS-CoV-2 infection, mice were infected with 3 × 10^8^ CFU Δ*agr* AH-LAC *S. aureus* or mock-infected with PBS intraperitoneally (IP). The *S. aureus* infection was performed via IP administration due to the technical difficulties of performing intravenous injections in a biosafety level 3 laboratory setting. Mice were monitored daily for weight loss and signs of disease. Disease score was based on weight loss, and the observation of reduced mobility, hunched posture, and ruffled fur. In some experiments, mice were sacrificed on day 4 post-SARS-CoV-2 infection to harvest their lungs, spleen, and kidneys.

### Viral titer and bacterial burdens

Whole organs were collected in Eppendorf tubes containing 500 µL of PBS and a 5 mm stainless steel bead (Qiagen) and homogenized using the Qiagen TissueLyser II. One fraction of the homogenates was serially diluted in PBS and spotted on TSA plates for *S. aureus* Colony Forming Unit (CFU) enumeration, a second fraction was frozen at −80°C for plaque assay, and a third was diluted 4× in TRIzol reagent (Invitrogen) and then frozen at −80°C for qRT-PCR. In some experiments, a lobe of the lung was fixed in formalin for histology analysis.

For plaque assay, Vero E6 cells were seeded at a density of 4.5  ×  10^5^ cells per well in flat-bottom 12-well tissue culture plates. The following day, media was removed and replaced with 100  µL of 10-fold serial dilutions of the virus stock, diluted in the infection medium. Plates were incubated for 1 h at 37°C. Following incubation, cells were overlaid with 0.8% agarose in DMEM containing 2% FBS and incubated at 37°C for 72 h. Cells were then fixed with formalin buffered 10% (Fisher Chemical) for 1 h. Agarose plugs were then removed, and cells were stained for 20 min with crystal violet and then washed with tap water.

For RT-qPCR, RNA was extracted from the TRIzol homogenates using chloroform separation and isopropanol precipitation, followed by additional purification using RNA columns according to the manufacturer’s instructions (Direct-zol, ZYMO research). RNA was reverse-transcribed using the High-Capacity cDNA Reverse Transcription Kit (Applied Biosystems). To assess viral titer, qPCR was performed using Applied Biosystems TaqMan RNA-to-CT One-Step Kit (Fisher-Scientific), 500 nM of the primers (Fwd 5′-ATGCTGCAATCGTGCTACAA-3′, Rev 5′-GACTGCCGCCTCTGCTC-3′) and 100 nM of the N probe (5’-/56-FAM/TCAAGGAAC/ZEN/AACATTGCCAA/3IABkFQ/−3’). qPCR reaction conditions were 48°C for 15 min followed by 95°C for 2 min, and by 50 cycles of 95°C for 15 sec, and 60°C for 1 min. Serial dilutions of *in vitro*-transcribed RNA of the SARS-CoV-2 Nucleoprotein were used to generate a standard curve and calculate copy numbers per mg of RNA in the samples.

### Cytokine profiling

Cytokine profiles were determined using the MILLIPLEX Mouse Cytokine/Chemokine Magnetic Bead Panel 32 PLEX kit (Millipore Sigma). In brief, sera from SARS-CoV-2-infected mice, *S. aureus-*infected mice, SARS-CoV-2 and *S. aureus* coinfected mice, or mock-infected mice were inactivated with UV-C (254 nm) treatment, with a power density of 4,016 μW/cm^2^, for 15 min at a distance of 3 cm to allow removal of the samples from the ABSL3 facility. Following the kit’s instructions, premixed magnetic beads that bind specific cytokines were incubated with the mouse sera (12.5 µL; 1:2 dilution), standards, background, and quality control samples in a black 96-well plate for 2 h at room temperature with shaking at 650 rpm. The plate was washed three times on a magnet, then incubated with detection antibody for 1 h at room temperature with shaking at 650 rpm. Streptavidin-phycoerythrin was then added to each well and incubated for an additional 30 min at room temperature with shaking at 650 rpm. The plate was washed two times on a magnet. The beads were resuspended in drive fluid and run on a Luminex MAGPIX Multiplexing System with xPONENT software. Calculation of cytokine concentration per milliliter of serum was performed by the software. Data were exported from xPONENT and imported into Graphpad Prism 9 software.

### Statistics statement

Statistical significance was determined using Prism 7.0 b, with Mann-Whitney test, Kurksal-Wallis test, log-rank (Mantel-Cox) test, unpaired two-tailed Student’s *t*-test, or Fisher’s exact test as indicated.

For patient data analyses, The Kaplan-Meier (KM) method was employed to estimate survival curves, with the Wilcoxon-Breslow test being utilized to compare the survival curves amongst different patient groups. *P* values for the data in Table 1 were determined by the Wilcoxon Rank-Sum test for continuous variables χ test for categorical variables.

## Data Availability

The assigned NCBI BioProject accession number for the genome sequences is PRJNA1123240.

## SUPPLEMENTAL MATERIAL

Supplemental Material mbio.01667-24-s0001.pdf Figures S1-S4.

ASM does not own the copyrights to Supplemental Material that may be linked to, or accessed through, an article. The authors have granted ASM a non-exclusive, world-wide license to publish the Supplemental Material files. Please contact the corresponding author directly for reuse.
