# Supplementary material for: SARS-CoV-2 infection predisposes patients to coinfection with Staphylococcus aureus
Source: mBio. 2024 Jul 22;15(8):e01667-24. doi: 10.1128/mbio.01667-24 (PMC11323729; doi:10.1128/mbio.01667-24)
Supplement: Supplemental Material — Figures S1-S4. [file mbio.01667-24-s0001.pdf]

SUPPLEMENTARY MATERIALS

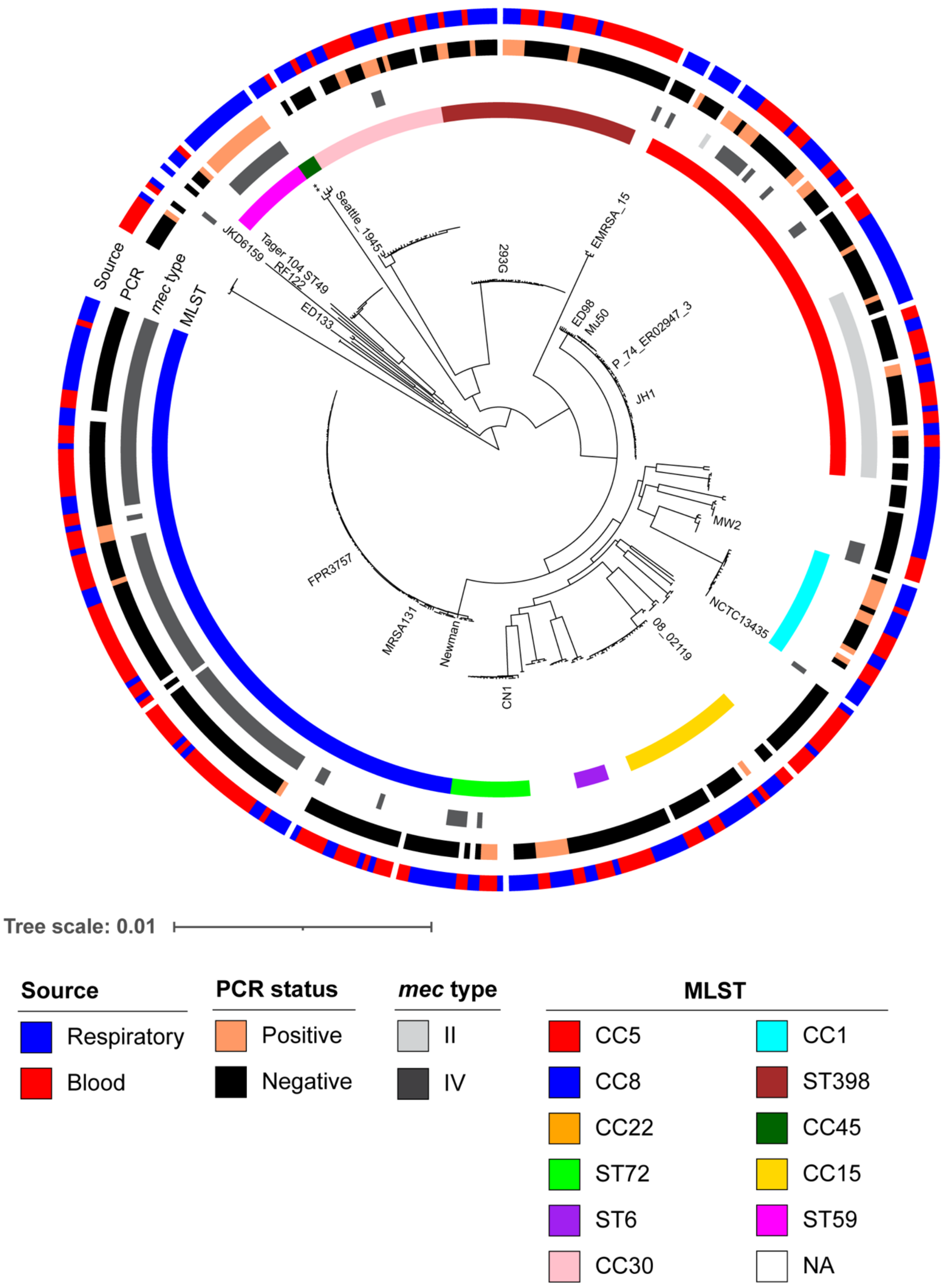

Figure S1. Phylogenetic analysis of *S. aureus* isolates.

All *S. aureus* isolates collected are indicated (including where multiple isolates were collected from the same patient at different times). Concentric circles represent CC/ST, mec type (Only type II and IV were represented in this collection of isolates. If no mec type is indicated, the strain is mec negative.), SARS-CoV-2 PCR result, and isolate source. ** = reference strain CA-347. CC = clonal complex, ST = sequence type, “NA” = other or not categorized.

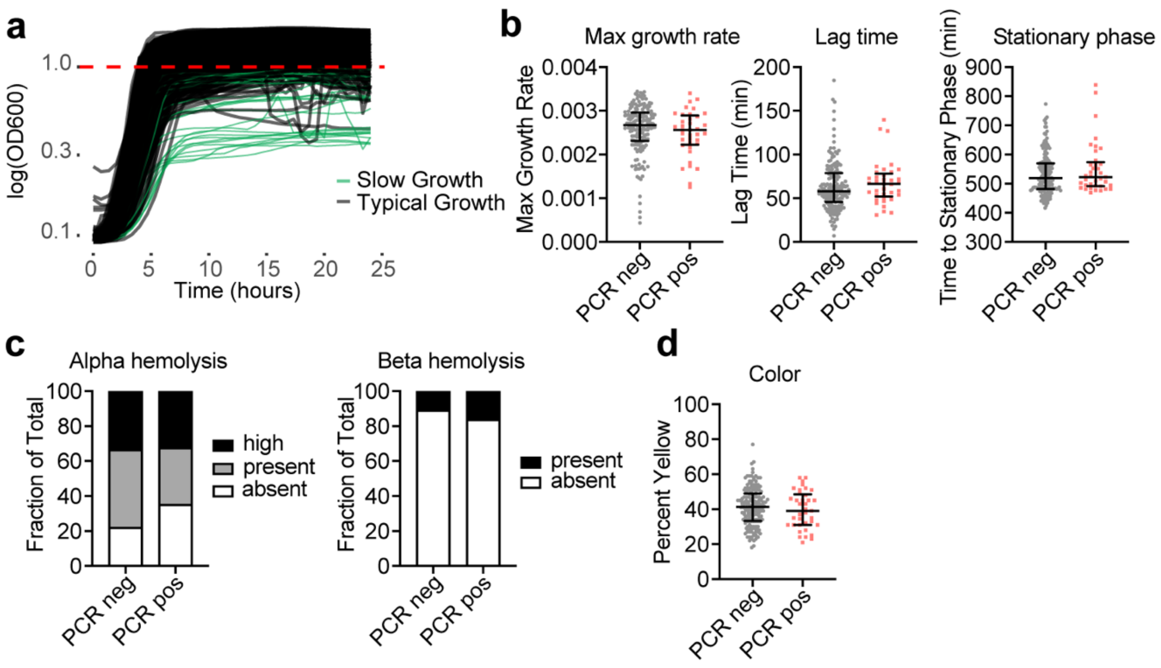

Figure S2. Growth, hemolysis, and color of the *S. aureus* isolates.

a. Growth curves of all isolates analyzed, with those that failed to reach an OD_{600} of 1 highlighted in green. **b.** Features of the growth curve of isolates from SARS-CoV-2+ and SARS-CoV-2- patients. **c.** Hemolysis of isolates from SARS-CoV-2+ and SARS-CoV-2- patients. **d.** Color of isolates from SARS-CoV-2+ and SARS-CoV-2- patients when plated on TSA plates ranking from dark yellow (100%) to white (0%). Error bars represent median with interquartile range. p> 0.05 for all plots, Mann-Whitney test (**b,d**), Fisher's exact test (**c**). PCR pos are isolates from SARS-CoV-2+ patients, and PCR neg are isolates from SARS-CoV-2- patients.

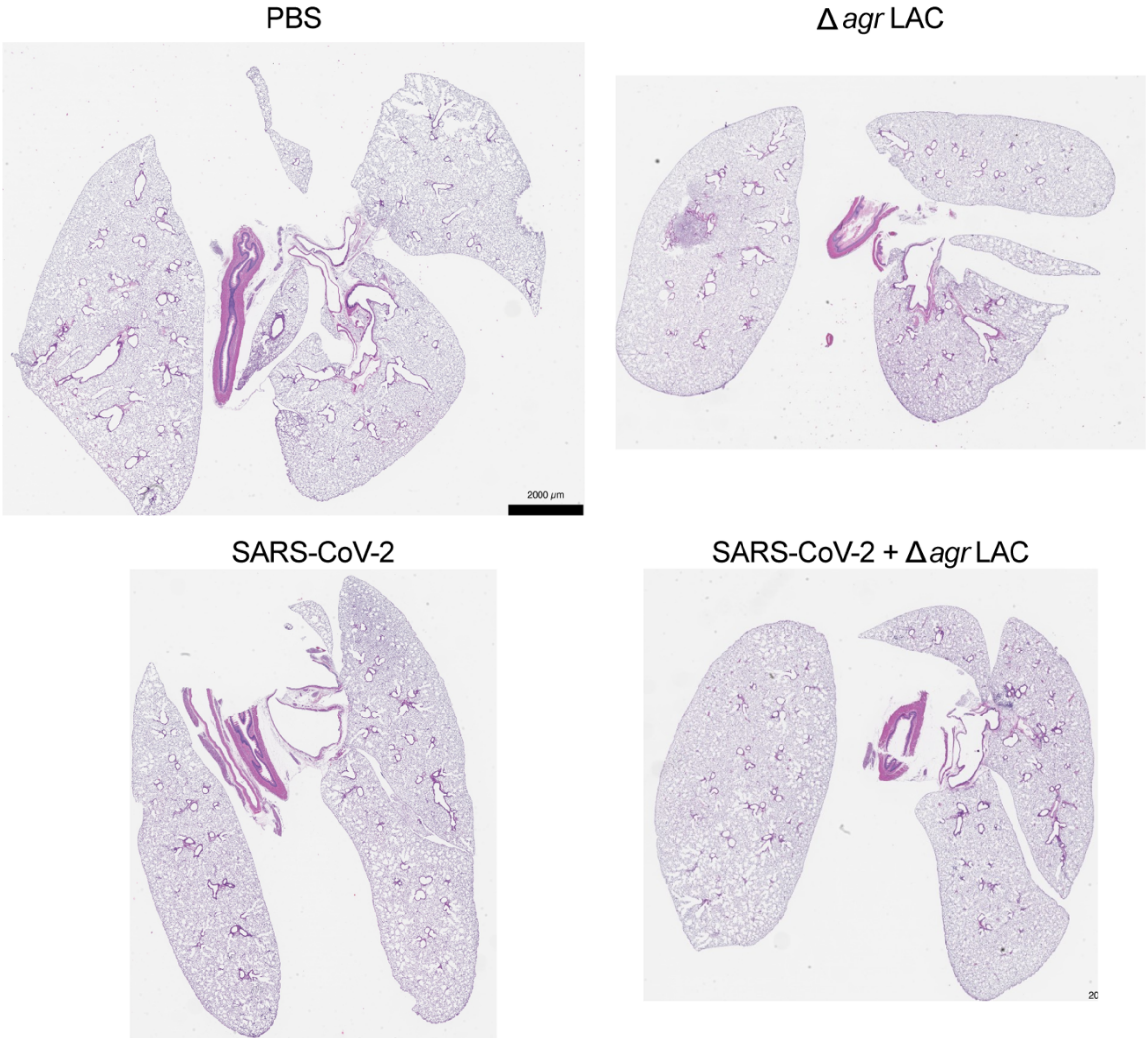

Figure S3. Histology analysis of co-infected lungs.

H&E staining of sections from lungs of mice infected with the indicated pathogens.

Representative images are shown.

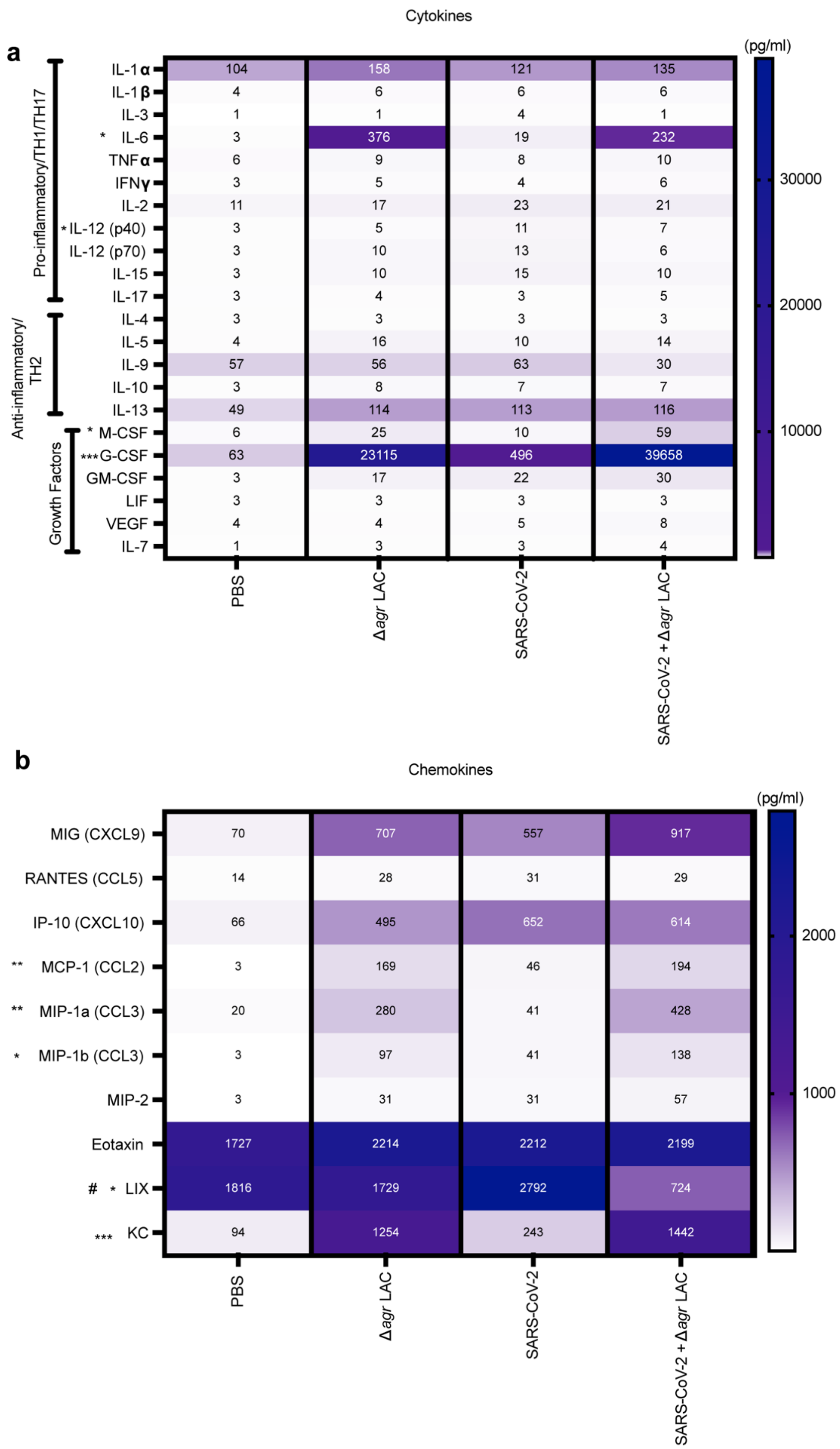
a
Cytokines
(pg/ml)
Pro-inflammatory/TH1/TH17
Anti-inflammatory/ TH2
Growth Factors
IL-1α 104 158 121 135
IL-1β 4 6 6 6
IL-3 1 1 4 1
* IL-6 3 376 19 232
TNFα 6 9 8 10
IFNγ 3 5 4 6
IL-2 11 17 23 21
* IL-12 (p40) 3 5 11 7
IL-12 (p70) 3 10 13 6
IL-15 3 10 15 10
IL-17 3 4 3 5
IL-4 3 3 3 3
IL-5 4 16 10 14
IL-9 57 56 63 30
IL-10 3 8 7 7
IL-13 49 114 113 116
* M-CSF 6 25 10 59
*** G-CSF 63 23115 496 39658
GM-CSF 3 17 22 30
LIF 3 3 3 3
VEGF 4 4 5 8
IL-7 1 3 3 4
30000
20000
10000
PBS
Δagr LAC
SARS-CoV-2
SARS-CoV-2 + Δagr LAC
b
Chemokines
(pg/ml)
MIG (CXCL9) 70 707 557 917
RANTES (CCL5) 14 28 31 29
IP-10 (CXCL10) 66 495 652 614
** MCP-1 (CCL2) 3 169 46 194
** MIP-1a (CCL3) 20 280 41 428
* MIP-1b (CCL3) 3 97 41 138
MIP-2 3 31 31 57
Eotaxin 1727 2214 2212 2199
* LIX 1816 1729 2792 724
*** KC 94 1254 243 1442
2000
1000
PBS
Δagr LAC
SARS-CoV-2
SARS-CoV-2 + Δagr LAC

Figure S4. Cytokine analysis of plasma from co-infected mice.

a-b. Cytokine **(a)** and chemokine **(b)** levels in serum at 4 days post infection. The mean of each group is shown. * indicates a difference between the SARS-CoV-2 infected group and the coinfected group, # indicates a difference between the *S. aureus* infected group and the coinfected group (Student's t-test). * $p < 0.05$, ** $p < 0.01$, *** $p < 0.001$.
